# 3D Cardiac Cell Culture: A Critical Review of Current Technologies and Applications

**DOI:** 10.3389/fcvm.2019.00087

**Published:** 2019-06-26

**Authors:** Christian Zuppinger

**Affiliations:** Cardiology, Department of Biomedical Research, Bern University Hospital, Bern, Switzerland

**Keywords:** 3D cell culture, induced pluripotent stem cells, cardiomyocyte, spheroid, engineered heart tissue, scaffold, high content screening

## Abstract

Three-dimensional (3D) cell culture is often mentioned in the context of regenerative medicine, for example, for the replacement of ischemic myocardium with tissue-engineered muscle constructs. Additionally, 3D cell culture is used, although less commonly, in basic research, toxicology, and drug development. These applications have recently benefited from innovations in stem cell technologies allowing the mass-production of hiPSC-derived cardiomyocytes or other cardiovascular cells, and from new culturing methods including organ-on-chip and bioprinting technologies. On the analysis side, improved sensors, computer-assisted image analysis, and data collection techniques have lowered the bar for switching to 3D cell culture models. Nevertheless, 3D cell culture is not as widespread or standardized as traditional cell culture methods using monolayers of cells on flat surfaces. The many possibilities of 3D cell culture, but also its limitations, drawbacks and methodological pitfalls, are less well-known. This article reviews currently used cardiovascular 3D cell culture production methods and analysis techniques for the investigation of cardiotoxicity, in drug development and for disease modeling.

## The Need for More Tissue-Like Cell Culture Models

Standard cell culture using adherent cells on multi-well plastic plates, dishes and flasks is an effective technique for expanding cell lines, bioproduction, and inspection of cells in defined conditions. However, as soon as cultured cells are expected to respond to drugs, toxins or signaling modifiers like *in vivo*, cell culture on flat surfaces, i.e., two-dimensional (2D) culture, turns out to be an imperfect or downright misleading (1–4). Cells in an intact tissue are embedded in extracellular matrix proteins (ECM) and are exposed to an abundance of biochemical, mechanical, electrical and other types of stimuli that lead to appropriate responses and fine-tuned changes in gene expression. In the heart, cells undergo cyclic deformation, show rapid calcium transients and electrical signals or experience shear stress from blood flow (1). In order to retain organotypic functionality as much as possible, a straightforward approach is to use fully differentiated cells directly isolated from living tissue, as these are considered to be in a native state (2). Animal models, explanted hearts and later freshly isolated primary cells have been used for the assessments of various parameters of cardiac cell physiology and electrophysiology for more than a century, which has increased our knowledge of the basic mechanisms of the heart tremendously (5–7). Large animal models such as minipigs or goats are also still needed as they provide the essential anatomical structures to study surgical interventions and to observe *in situ* clinically relevant pathophysiological changes of heart and vessels, such as the response to pressure overload, myocarditis or atherosclerosis and infarcts (3). However, animal models are relatively expensive, need experienced personnel, long-term housing, strict quality control, and there are ethical concerns (4). Also, there are differences between the human heart and those of animals, and these differences can become more pronounced and limiting in pathological conditions (8). For these reasons, it would be advantageous to have access to *in vitro* screening models of the myocardium that allow the study of long-term effects of drugs, environmental factors and gene mutations, preferentially on a human genetic background.

**Figure 1:**
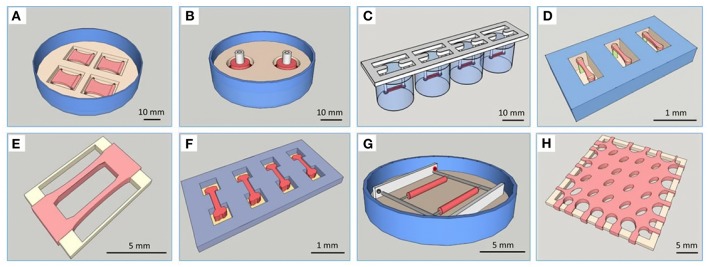
Overview of different types of EHTs. Reprinted with permission from Weinberger et al. (11). **(A)** Plane EHT on Velcro-covered rods (5), **(B)** fabrication of ring-shaped EHTs (12), **(C)** fibrin-based mini-EHT on polydimethylsiloxane (PDMS) racks (13), **(D)** cardiac micro tissues (CMT) on fluorescent pillars (14), **(E)** cardiobundles on a PDMS frame (15), **(F)** micro heart muscle (16), **(G)** cardiac biowires (17), **(H)** cardiac patch (18).

**Figure 2:**
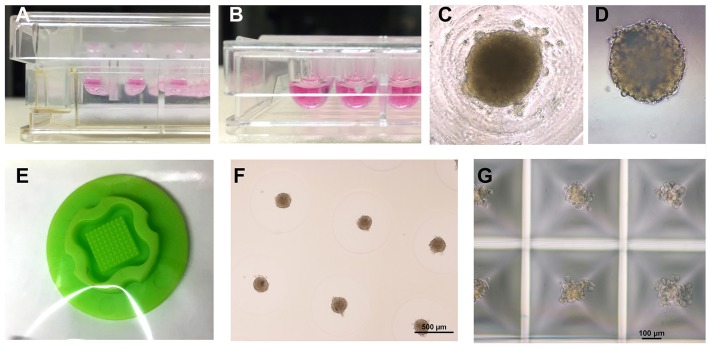
Overview of different spheroid production methods. **(A)** Hanging drops (InSphero, GravityPlus), **(B)** U-shaped multi-well plate with non-adhesive coating (Greiner bio-one, Cellstar), **(C)** view inside a U-shaped well at day 2 of culture, **(D)** Spheroid made in hanging drop for 3 days was transferred to a non-adhesive multi-well plate for further culture (InSphero, GravityTrap), **(E)** Silicone micro-mold (3D Petri-dish, microtissues.com) for making an agarose cast for 81 spheroids, **(F)** Cardiac spheroids reside inside the 3D petri-dish agarose cast submerged in medium 4 days after seeding of the cell solution, **(G)** Small cardiac spheroids forming 3 days after seeding in a micro-patterned multi-well plate with 12 × 750 microwells (Kugelmeiers, Sphericalplate 5D). All photos by the author.

Because of the above-mentioned limitations of classic models, three-dimensional (3D) culture systems have been developed that attempt to restore *in vivo* conditions in some sort of a multicellular micro-tissue (MT) with and without additional, natural or synthetic biomaterials, also called scaffolds. Historically, 3D cultures have first been used in a systematic manner for drug testing in cancer biology, which is explained in part by the fact that cellular aggregates with a hypoxic core show many similarities with avascular solid tumors (9). It has been repeatedly found, that only 3D technologies using co-cultures are able to mimic key aspects of the phenotypical and cellular heterogeneity as well as microenvironmental aspects of tumor growth (10). In the cardiovascular field, current 3D cell culture model systems that are in use for drug testing and toxicology applications mostly fall into two main categories: They contain A. a scaffold matrix, typically a hydrogel, which is mixed with and populated by cells and forms a strip or hourglass-shaped contracting MT between attachment sites, also called an engineered heart tissue (EHT) (Figure 1) (5), or B. smaller cellular aggregates (spheroids) forming by self-assembly without scaffold proteins in hanging drops or in multi-well plates with non-adhesive surfaces (Figure 2). An increasing number of studies making use of cardiac scaffold-free spheroids for drug testing and toxicology has been published in the last couple of years (6, 7) and frequently, a mix or co-culture of several cell types is used, such as rodent or human primary- or human induced pluripotent stem cell-derived cardiomyocytes (hiPSC-CMs), fibroblasts, stem cells, and endothelial cells (19–23). Additionally, microfluidic systems, micro-patterns, and microphysiological platforms, including various sensors, pumps and perfusion, and other technologies have been developed around the living components of such model systems (24). Larger tissue formats like multilayered cell sheets, re-cellularized hearts or large biomaterial patches usually are too expensive and slow in the making for drug screening purposes and are instead developed for regenerative medicine (25). Recently, the term “organoid” is more often used in the literature. However, this term should not be used for every 3D cell culture as it implies, at least by the original definition, self-organization of stem cells that leads to differentiated organotypic structures and functionality (26). In the cardiovascular field, self-organization *in vitro* has been observed in vascular networks (27), but for cardiac muscle cells, it is mostly limited to self-assembly of clusters by aggregation, alignment, concerted contractions and some degree of cellular maturation, while the actual embryonic development of a vascularized organ with chambers, working pump function and conduction system currently is not feasible to replicate *in vitro*.

## The Cellular Components of Cardiac 3D Culture and Disease Models

The availability of differentiated cells for research is a challenge in the cardiac field as postnatal mammalian cardiomyocytes do not proliferate, and primary human cells are not available in significant quantities (28). The use of primary ventricular cardiomyocytes from adult animals or patients is not recommended for 3D culture as these rod-shaped cells do not integrate well in spherical aggregates and many cells become necrotic when kept floating for longer time (own observation). Fortunately, the development of hiPSC-CMs has helped to improve this issue and promoted research with human cardiomyocytes and 3D models in the cardiovascular field, even if the currently available hiPSC-CM are relatively immature (28–30). Also, the cell population is not entirely uniform as ventricular-type, nodal and atrial-like action potentials are found when single cells are analyzed using electrophysiological methods (29). Furthermore, there is heterogeneity in the expression of cytoskeleton and sarcomeric proteins such chamber-specific myosin light chains or troponins, and different grades of structural organization of sarcomeric proteins has been observed (30, 31). Another practical issue encountered with current protocols is the inconstant efficacy of the differentiation process and batch-to-batch variations that lead to baseline variations when cells from the same patient are repeatedly re-programmed (11, 32). The incomplete maturation of the cells may reduce the predictive power of the model system, considering that cardiovascular diseases predominantly occur in the elderly human population, although this demerit is not limited to hiPSC-CMs (33, 34). Several strategies, particularly with 3D cultures, have been employed to enhance the maturation of cultured hiPSC-CM such as novel iPSC reprogramming methods, changing the energy sources in specialized media, finding the ideal developmental time window for experiments, and electric and mechanical training of EHTs (35–38).

With the technology of hiPSC-CMs arrived the option of using patient-derived cell lines with disease-specific phenotypes and known mutations on a human genetic background, and with the full knowledge of the patient's medical history. This is an exciting prospect as it might enable new options in personalized medicine and gene therapies *in vitro* (39, 40). Similarly, hiPSC-CM technology has been used to model a number of inherited heart diseases, among them are Duchenne muscular dystrophy, Fabry disease, Danon disease, familial hypertrophic cardiomyopathy and others (41). Many of these hiPSC cell lines are publicly available in stem cell banks for use in different model systems including 3D culture approaches (42). The hypothesis that 3D culture models can provide tissue-like features was supported by using hiPSC-CMs with a disease-specific genotype in an EHT-model: The contractile deficit of hiPSC-CMs with a truncation in the sarcomeric protein titin was not visible in 2D cultured cardiomyocytes but became obvious in EHTs working against the elastic resistance of PDMS or silicone pillars (39). Regarding disease models, applications in the field of cardio-oncology have motivated our lab to explore different cardiomyocyte model systems from primary adult rat cardiomyocytes to monolayers and cardiac spheroids made of hiPSC-CM and to study cancer therapy-associated changes of contractility and calcium handling (40, 43–45). Other cardiomyopathies that have been modeled in 3D cultures include cardiac fibrosis (46), hypertrophy (47), Chagas disease (48), atrial fibrillation (49), cardiotoxic cancer therapies, and other toxins (19, 20, 22, 44, 50).

## Production Methods

A number of different types of 3D culture models and production methods have been developed in the cardiovascular field (Table 1). The most basic form of 3D culture is the multicellular aggregate as it occurs by self-assembly of floating cells on low-attachment surfaces (also called liquid-overlay method). Such a suspension culture can be made in inexpensive ways, for example by using a sterile dish with a thin film of agarose. Spontaneous formation of this type of multicellular aggregate has already been observed at the time of early cardiomyocyte isolations from fetal or newborn animals (57). Many suppliers of cell culture products make variants of U-shaped bottom multi-well plates with special coating for ultra-low attachment that leads to self-assembly of spheroids (Figures 2B,C), or special formats for the mass-production of small aggregates (Figure 2G). Similarly, soft silicone molds can be used to make agarose-casts with many small wells for the production of microtissues (Figures 2E,F). The hanging drop technique has allowed producing uniform microtissues in a reliable way (44, 55), and sophisticated systems have been developed with hanging drops as part of microfluidic systems including perfusion and sensors (56). Overall, an advantage of the spheroid as 3D cell culture is the option of using semi-automatic methods to produce spheroids by using a pipetting robot for filling multi-well plates, exchanging medium, drug treatments, and finally analyzing the samples in high-content readers. Advantages and disadvantages of the spheroid culture vs. the EHT-models are listed in Table 2. The EHT model was conceived in the 90ies for the purpose of tissue engineering (5). Soon it was also used for drug testing and disease models with the option to measure contractile force, either directly or by the deflection of silicone poles, as well as calcium transients and electrical signals (13, 32, 61). Variants of the EHT model were developed that have in common the casting of a hydrogel, usually containing fibrin/thrombin and/or collagen and Matrigel components, with the addition of either primary newborn rodent cardiomyocytes or stem-cell derived cells as single and co-cultures and different geometries and analysis options (Figure 1). Elastic silicone posts deflect with the contractions and allow the tissue to contract auxotonically and perform contractile work, the physiological form of cardiac contraction (11). Smaller formats were developed for the purpose of drug treatment and optimization of maturation protocols (38, 41), while larger formats are suitable for regenerative therapies (11).

**Table 1:** Overview of cardiovascular 3D cell culture technologies.

**Type**	**Specific bio-functional properties**	**Companies**	**References**
Molded natural or synthetic hydrogels populated by cells, attached to elastic micro-poles or a frame	Microtissues showing linearly aligned cardiomyocytes and improved tissue and cellular maturation after training, force measurements are feasible	EHT-technologies, novoheart	(5, 12, 13, 15, 17, 38)
Enclosed cells and hydrogels (in molds, tubing, or microfluidic channels), with perfusion	For microvascular models, self-organizing cells, may include shear-stress and stretch, measurement of barrier function	Mimetas, AlveoliX, TissUse	(51–54)
Self-assembling multicellular aggregates on low attachment plates, hanging drops or micropatterned surfaces	Small aggregates and spheroids showing spontaneous beating activity, can be mass-produced, treated and analyzed by semi-automatic systems	InSphero, microtissues.com, Corning, Kugelmeiers, Stemcell, Greiner, Nunclon, Cytoo	(6, 44, 55–57)
Magnetic levitation, bioprinting of larger structures, layering of sheets	Large tissues consisting of different cell types and biomaterials, making entire organs as the ultimate goal	n3D Biosciences, RegenHU, CellInk, Biolife4D	(58–60)

*Literature references and companies represent a non-exhaustive list and the author apologizes for any omissions*.

**Table 2:** Comparison of advantages and disadvantages of scaffold-based models and cardiac spheroids.

**Advantage**	**Features of cardiac spheroids**	**Disadvantage**
Uses only a small number of potentially costly cells per data point	Small size of multicellular aggregates	Methods like protein chemistry and RNA extraction need pooling of spheroids
No interference of scaffold proteins with the development of the microtissue or the outcome of assays	Made without additional scaffold proteins	Some ECM factors could improve survival and self-organization of the tissue
Spheroid is quickly formed and ready for drug treatment and analysis	Assembles spontaneously by gravity or on non-adhesive surface	Little control over the distribution of cell types or overall shape, may result in multiple spheroids
Manipulation by pipetting and sedimentation, no touching	Spheroids are floating in culture	Spheroids may get lost or stuck on surfaces in pipetting steps
Mimics thicker tissues (and tumors)	Larger spheroids develop zones of o_2_, PH, metabolites	Reduced viability, variable results, limited diffusion
Motion activity and calcium cycling correlates with cell viability and drug treatment	Show long-term spontaneous contractions	No direct force measurement, non-linear cell alignment
Miniaturized multi-well formats and compatible with plate readers	Spheroids can be cultured in single wells	-
**Advantage**	**Features of scaffold-based models (EHT)**	**Disadvantage**
Shape can be tailored for applications (screening, maturation, regen. Medicine)	Shape is determined by the scaffold/hydrogel mold	Uses large number of cells per tissue
Hydrogel can be adapted for organotypic functions and pathologies (vascularization, stiffness)	Made with scaffold biomaterial and ECM proteins	Limited diffusion, risk of breaking, unequal distribution of cells, potential interference with assays
Sensors can be integrated in microphysiological devices	Tissues attached to support structures	Manual steps necessary, small number of tissues of the same batch of cells
Mech. And electrical training, physiological function in disease models, force assessment	Linear alignment of muscle cells	-
Technology development toward tissue engineering applications	Training protocols show improved maturation of hiPSC-CM	-

Vascular *in vitro* models have improved in recent years with the advent of microfluidic systems and the hope is, that these systems may partially replace animal experimentation that has been common in this field of research (62). Current vascular *in vitro* models apply different molding techniques, bioprinting, and combinations of these technologies for producing micro vessels on organ-on-chip platforms (51–53, 59). The challenge of oxygen supply in larger artificial tissues has been a matter of active research in tissue engineering for some time (24, 63). However, establishing a perfused vascular network *in vitro* turned out more difficult than initially anticipated since these processes are inherently multi-factorial and require a fine-tuned expression and post-translational processing of growth factors, a complex spatial localization of angiogenic signals in the ECM, and the collaboration of multiple cell types (organ-specific endothelial cells, pericytes, vascular smooth muscle cells) (64, 65). Instead of relying on cellular self-organization for establishing vascular networks, recent studies rather use pre-formed channels or bioprinting approaches to reach this goal (60). Additional concepts have been published that are making use of combined techniques, such as cell layers with pre-formed vascular trees obtained from animals, bioprinted and microcontact models as components of micro-physiological platforms and larger tissues for surgical applications (51, 66, 67).

## Analysis Methods

For endpoint analysis, classic lab methods like tissue fixation, paraffin embedding, histology, cryosectioning, immunolabeling, and cell viability/cytotoxicity assays are feasible with most types of 3D cultures where the cells are accessible. Protein chemistry, RNA isolation and histology usually require pooling of groups of smaller cellular aggregates (own observations). A number of cell viability/toxicology assays are commercially available that can be performed with either live MT or lysed material (67). Cell physiology methods for the investigation of cardiac features in living tissues such as contractions, force, calcium cycling or electric signals require specialized instrumentation depending on the sensitivity of the sensors and the desired temporal and spatial resolution (68). Although methods exist to directly measure contractile force in single cardiac cells and small muscle strips, these methods require a skilled workforce in order to provide good reproducibility and have a slow throughput (69). Instead, a variety of optical methods have been developed to measure length changes of the whole cells or sarcomers during the contractile cycle of mammalian cardiomyocytes (70–72). These video-based systems are either commercially available as complete bundles of hardware and software (IonOptix, Sony, EHT technologies), or as open access software for image analysis that can be used with existing microscopes and cameras (73, 74). Such a video-based analysis can be realized in inexpensive ways using modified consumer cameras (so-called action cameras with high frame rates up to 240 frames per second) and open source software (68). Optical measurement using white light has the advantage of being label-free and non-invasive, so the measurement can be repeated many times, even while the cell culture is in the incubator if a camera is placed inside an atmosphere- and temperature-controlled environment (68). Besides classic electrophysiology methods using patching and impaling with sharp microelectrode pipettes, multi-electrode arrays, and impedance spectroscopy have been used to measure electrical signals and contractile activity in cardiac 3D models (75–78). Calcium-binding fluorescent dyes and voltage-sensitive dyes have been used in 2D- and 3D-cultures as this method allows to measure a greater number of samples in relatively short time, and for some applications high-content readers can be used for this purpose (Hamamatsu Photonics, Molecular Devices, PerkinElmer) (50, 79). Although these optical methods provide good results for live 3D cultures as a whole tissue, obtaining data at (sub-)cellular level from inside these MT is challenging without relying on time-consuming histology methods. High optical resolution comes at the price of limited penetration depth and technical issues such as the permeability for fluorescent dyes and antibodies, the working distance of lenses and the geometry of sample holders limit these whole-mount microscopy applications. Recently, several approaches to “clearing” MTs (i.e., homogenizing the refractive index of the fixed tissue so that it becomes transparent) have been published, and commercial solutions have become available, especially for the use with spheroids and fluorescence high-content confocal imaging (80).

When it comes to choosing a 3D culture model system, the requirements of the project dictate crucial analysis methods. For a study of mechanical features of a muscle-construct, a hydrogel-based EHT model system might be chosen, where the cells experience mechanical load and have the ability to align longitudinally. Contractile force can then be measured either directly or using video methods by measuring the deflection of attached micro-posts or pillars of known strength (39, 40, 49, 50). Otherwise, if mainly the spontaneous or electrically paced beating pattern and viability of cardiac tissues is of interest in a larger number of samples, cardiac spheroids may be the model of choice. Cardiac functionality of these spheroids can be analyzed A) using label-free methods either by computational video analysis or electrical impedance spectroscopy methods, or B) using calcium- or voltage-sensitive dyes in multi-well plate readers, or by using more advanced microscope equipment for multiparametric assessment (19, 44, 56, 68, 81–84). A comparison of features and comparative advantages or disadvantages of these scaffold-containing or scaffold-free models is shown in Table 2. Finally, it could be summarized that spheroid models are easier to integrate in existing drug developmental pipelines and to upscale the number of tests in the same batch, while the ETH models provide a better physiological representation of the myocardium and thereby enable the analysis of advanced disease models.

## Why is 3D Cell Culture Not Used More Often?

Although many tools and reagents for making and analyzing 3D cell culture models are commercially available, and the number of publications in all fields of life science is increasing (85), the technology is infrequently used, outside of regenerative medicine, in academic research labs or industry several decades after the publication of the first studies (86). Reasons for this situation include that standard 2D culture is well established, with ample literature available, and previous studies to compare results (87). Furthermore, 2D culture has gained uncritical acceptance in the past, is less expensive, is more standardized, and is often easier and less time-consuming to analyze and to handle in the lab. When considering practical aspects of working with 3D culture, seemingly trivial tasks such as regular checking for culture health and growth are more difficult with most 3D models, because even smaller tissues usually are opaque and single cells not discernable unless stained (Figures 2C,D). Additionally, manual handling of the microtissues and culture medium can be challenging when the MTs are free floating, fragile or access to the tissue(s) is obstructed by surrounding containers and technical equipment. Some systems facilitate the handling of spheroids by trapping them in conical wells, in perfused chambers inside organ-on-chip designs or by incorporation of magnetic nanoparticles (88, 89) (Table 1). Fortunately, there are more technologies and products coming to the market that are targeted at 3D cell culture applications such as different formats of spheroid and EHT production systems, microfluidic technology and suitable ready-to-use equipment, reagents for the clarification of thicker tissues and adapted microscopes and software for live imaging. Finally, and despite the above-mentioned technological advancements, the research community has to agree to a set of standards and read-outs to use in efficacy and toxicity screening (90). Replacement of animal research by *in vitro* technologies using human cells is another substantial incentive for 3D cell culture, and already had a significant impact, for example, on how cosmetics are tested *in vitro* as the use of animals is banned for this purpose in Europe (4, 67). Still, it can be debated if existing 3D models already provide sufficient evidence for superior predictions of the clinical outcome of a new drug, and if these models show enough physiological relevance compared to animal or human tissue, or still lack complexity, for example regarding the role of the vasculature or the immune system (12, 90).

## Conclusions and Outlook

Each *in vitro* model has advantages and disadvantages for using it with certain assays, regarding organotypic features and production methods. Therefore, it needs to be decided upfront, which parameters and organotypic features are essential to be included in the study and if going 3D is making sense in the context of a particular project. Apparently, if the project deals with features of single cells isolated from its myocardial environment, then 2D cultured cardiomyocytes offer the option to use classic electrophysiology, high-resolution microscopy, and other methods. Instead 3D culture mimics features of larger tissues or entire organs and is the method of choice for co-culture models. 3D cardiac cell culture technologies hold great potential for applications in tissue engineering, drug development, cardio-toxicology and disease modeling. But a considerable effort is still needed to assure the accuracy, relevance, and reproducibility of these models and to improve automation and readout techniques. Instead of pushing many different systems to the market, careful analysis of core concepts may be instrumental for establishing 3D cell culture as a widespread and validated tool in life science.

## Author Contributions

All content is prepared by the author CZ with the exception of Figure 1 that is reprinted with permission from Wolters Kluwer Health, Inc.

### Conflict of Interest Statement

The author declares that the research was conducted in the absence of any commercial or financial relationships that could be construed as a potential conflict of interest.
